# “‘The Tragedy of the Punch Drunk': Reading Concussion in Australian Sporting Newspapers, 1843–1954”

**DOI:** 10.3389/fspor.2021.676463

**Published:** 2021-07-15

**Authors:** Stephen Townsend

**Affiliations:** School of Human Movement and Nutrition Sciences, University of Queensland, Brisbane, QLD, Australia

**Keywords:** sports related concussion, history, Australia, newspapers, media analysis, concussion, brain injury - traumatic, distant reading and close reading

## Abstract

Australian cultural attitudes toward sports related concussion (SRC) are understudied. Australia has a long history of valorising combat, collision, and contact sports, in which SRC is a common occurrence. It is therefore vital to understand how sociocultural and historical factors shape Australian attitudes toward SRC, in order to more critically evaluate the decisions made by athletes, parents, coaches, and others with regards to risk and brain injury in sport. This paper analyzed historical representations of SRC in Australian sporting newspapers between 1803 and 1954. Using distant reading, this analysis revealed four distinct periods of increased press discourse about “concussion,” which were subject to interrogation *via* close reading. Close reading revealed that concussion was being reported in the Australian sporting press as early as 1859. Further analysis revealed critical and scientifically informed discussions about the delayed effects of concussion in 1901, systemic critiques of sporting organizations' response to concussion in 1906, and evidence of a limited concussion crisis in Australian boxing during the early 1930s. The findings of this research show that concussion was not only being reported in Australian newspapers throughout the late nineteenth and early twentieth centuries but it was subject to critical and informed commentary that has striking similarities with current debates about SRC. Despite this, widespread systematic changes to Australian sport did not occur until recently. This raises important questions about the political and institutional factors that prevented a major concussion crisis from developing in Australia during the early twentieth century, and prompts us to further consider the distinguishing features that facilitated the development of the current crisis.

## Introduction

On the evening of October 24, 2020, I was in a crowded pub in Meanjin (Brisbane), Australia^1^. Seated around me was a collection of my closest friends, and there was a large screen hanging from a wall near our table, onto which was projected a football game. There was a sense of excitement around the room, more than the usual hum that preceded an Australian Football League (AFL) ^1^ Grand Final. For some of us, it was our first time in a bar since the Coronavirus disease (COVID-19) lockdowns were eased and the novelty of being out in public was further amplified by the knowledge that many other people around the world were confined to their homes by the pandemic. The crowd was also energized because the game was being played in our home town for the first time in its history, having been relocated from its traditional home in Melbourne to avoid a disease outbreak in that city. The location change disrupted a 118-year tradition of the Grand Final being played at the Melbourne Cricket Ground, and it also had more tangible effects on the day's proceedings. The Grand Final is usually played under mild spring sun in Melbourne, where dry conditions favor the skillful ball handling and precise kicking that make Australian Rules football a pleasure to watch. By comparison, Meanjin is a subtropical moisture trap at that time of year, and there was heavy rain falling throughout the game. This tends to exacerbate the most violent aspects of Australian Rules football. Rain turns the leather ball into a slippery cake of soap and, as players fumble after it, they are exposed to increased risk of being tackled, which can happen from any direction and at almost any time. Instead of the ordered battle lines that characterize American football or the Rugby codes, gameplay in Australian Rules football looks more like a series of isolated melees. Players roam the field freely and compete for possession of the ball through what sociologist Jay Coakley calls brutal body contact: tackling, bumping, or body checking their opponents (Coakley, 2017, p. 142). The collisions at these junctures are rapid, unpredictable, and sometimes jarringly violent.

In the sixth minute of the Grand Final, Richmond's Nick Vlastuin and Geelong's Patrick Dangerfield sprinted toward one another from opposite sides of a bouncing ball. Vlastuin appeared to reach the ball first but recoiled momentarily when he saw Dangerfield's cocked elbow racing toward him. Vlastuin crumpled as Dangerfield's forearm crunched into his jaw and as his unconscious body slumped to the turf, the umpire frantically whistled a halt to play. It was immediately clear that he would not wake for some time. While he received medical attention on the ground, the commentators offered their various interpretations of the collision, with veteran caller Bruce McAvaney calling it “unimaginable^2^.” It was not unimaginable to the officials, the fans, or the players involved. Vlastuin's concussion was a predictable consequence of hypercompetitive contact sport. There was no on-field penalty for Dangerfield and he received no sanction after the game. He told the media that he “did not see much in it … for everyone, it is just play on. It is a contact sport^3^.” Vlastuin was similarly unperturbed by his injury, despite not being able to remember what month it was when he eventually regained consciousness. He told reporters “it is just one of those things in footy (see text footnote 3).” He is well-qualified to make such an assessment; the Grand Final concussion was his fourth in 3 years^4^.

The people around me reacted in a variety of ways. Some cheered the carnage, some winced and looked away, some reenacted the collision with their friends, and some stared quietly at the prostrate figure on the screen. I think those in the latter category might have been thinking the same thing as me; this *is* a normal part of Australian sport … but for how much longer? Like many other countries with commodified sporting cultures, Australia is grappling with “the concussion crisis in sport,” which Dominic Malcolm believes is “calling into question the long-term future of some of our most well-established sports (Malcolm, 2020, p. 2).” The crisis is driven by concerns about the potentially devastating neurological consequences of sports-related concussion (SRC)^5^. Although athletes like Vlastuin and Dangerfield may view concussion as an acceptable risk of their profession, many other Australians are reevaluating their attitudes toward head injuries in sport, exercise, and leisure activities. This shift must be understood in terms of the biomedical discoveries that have prompted it and also the cultural discourses that shape how Australians perceive health, risk, and injury in sport.

In doing so, this study engages with an emerging field of research that interrogates the concussion crisis from sociocultural perspectives, and is guided by the medical humanities tenet that “biomedicine does not hold all the keys to understanding the experience of illness (McNaughton and Carel, 2016, p. 294).” By interrogating historical press perspectives of SRC in Australia, this study challenges the Australian and international tendency to view concussion almost exclusively “through a biomedical or neuroscientific lens (Ventresca and McDonald, 2020, p. 4).” Biomedical research is vital for understanding the neuropathology of SRC, and future advances may help to mitigate its negative health consequences. However, it is not clear when or if these advances might eventuate. In the absence of biomedical or technological solutions for SRC, current strategies primarily focus on modifying the behavior of athletes through rule and policy changes, education programs, and some limited attempts at cultural change (Gunasekeran et al., 2020). The efficacy of these strategies can be increased if athletes, parents, coaches, officials, and policymakers are able to recognize and understand the “social, economic, political, and historical forces” that influence perceptions of brain injury in sport, exercise, and leisure activities (Ventresca and McDonald, 2020, p. 4). Put more simply, addressing the concussion crisis requires us to understand how cultural attitudes shape the decisions that athletes make about their brain health. This analysis of historical representations of SRC in Australian sporting newspapers contributes to a more critical understanding of the more than medical forces that continue to shape twenty-first century approaches to concussion.

## The Concussion Crisis in Australia

Recent concerns about SRC in Australia were prompted by international developments, most notably the 2005 diagnosis of Chronic Traumatic Encephalopathy (CTE) in the deceased National Football League (NFL) player Mike Webster. This diagnosis built on more than a century of brain injury research but appeared revelatory to much of the press and public, and prompted widespread debate about the health risks of collision sports like football (Solomon, 2018). Although these initial diagnoses were specific to the USA, it did not take a great leap of imagination to suppose that athletes in other collision sports and other countries might also be at risk of developing neurodegenerative diseases. Combat, collision, and contact sports are extremely popular in Australia. In 2019, they constituted five of the top 10 most participated sports for Australian boys, and three of the top 10 for girls^6^. Other popular activities, such as cricket and gymnastics, for Australian children also pose concussion risks^7^. Australians participate less in organized sports as they enter adulthood but sports, especially football, retain major cultural significance. The AFL is the fourth most attended domestic sporting league in the world, and, for the last 5 years, the Grand Final has been the most viewed Australian television program each year^8^. In 2019, six of the top 10 most popular Australian television programs were either Rugby League or Australian Rules football matches (see text footnote 8). Gameplay in these codes is violent by design and regularly results in concussions^9^. For example, a 2020 study of National Rugby League (NRL) players found that 67.2% of the players had sustained at least one concussion in the previous two seasons (Longworth et al., 2021).

In 2019, the Government of Australia dedicated $50 million AUD over 10 years toward researching traumatic brain injuries with a specific focus on “concussion while playing sport^10^.” The Australian Sports Brain Bank was founded in 2018 and, in the same year, Sport Australia (the peak federal government body responsible for sport), released its ‘Concussion in Sport' position statement, which notes that ‘sport-related concussion is a growing health concern in Australia' with ‘elevated public awareness' (Elkington et al., 2019). In 2021, the AFL announced that it was working to establish a 2-billion-dollar trust that would provide former players with care and neurological support^11^. Australian media interest has also intensified. A Google search for Australian news stories shows that after rising steadily from 2010 onward, the number of articles published about “concussion and sport” spiked dramatically in 2019 and 2020. In 2019, the number of articles (3120) written about SRC doubled from the previous year and more than doubled again in 2020 (7120)^12^. This quantitative increase in reporting was punctuated by a number of lengthy investigative pieces, including an article published in the *New York Times* that showed SRC in Australian sport was subject to both international and domestic scrutiny^13^. Media interest intensified further when the first Australian cases of CTE were announced. In mid-2019, Associate Professor Michael Buckland's team at the Australian Sports Brain Bank diagnosed the disease in two unidentified former professional Rugby League players^14^. More sensational diagnoses came in 2020 and 2021 when they found CTE in the brains of the deceased Australian Rules icons Graham “Polly” Farmer, Danny Frawley, and Shane Tuck. These diagnoses forced the Australian public to confront a reality that was perhaps most succinctly expressed by Buckland: “CTE is real, and it is here in Australia^15^.”

## Distant Reading SRC in Australian Newspapers

The intense interest in SRC in 2021 raises questions about how this issue was represented by the Australian press in the past. This not only provides context and scale for the current crisis but also reveals insights about the antecedents of twenty-first century attitudes toward SRC. Concussion has always been a feature of sport, and medical authorities have been voicing concerns about its potential neurological dangers since the late nineteenth century (Casper, 2018, p. 798; Stone et al., 2014). In the USA, fears about concussions in collegiate football received such widespread and intense media coverage in the late nineteenth and early twentieth centuries that historian Emily Harrison argues America's “first concussion crisis” occurred as early as 1906 (Harrison, 2014). The extent to which early concerns about SRC were publicly debated in other national contexts is less well-known. One of these understudied national contexts is Australia, a nation with a long history of valorizing combat, collision, and contact sports. This study takes a first step toward bridging this knowledge gap by analyzing one aspect of SRC history in Australia: its historical representation in sporting newspapers. By combining distant and close reading methodologies, this study produces a series of vignettes that reveal changes to Australian newspaper representations of SRC over time.

The sources for this analysis were provided by Trove, an online multimedia archive administered by the National Library of Australia (NLA). In addition to letters, diaries, official records, and a range of other historical sources, Trove hosts a collection of 1,629 newspapers and gazettes that spans multiple geographic, political, and ideological perspectives. Trove is a valuable resource for historians but it does have limitations. Arguably the most significant of these is the 1,954 cutoff in coverage due to Australian copyright law. The curtailing of Trove's coverage in 1954 hampers an examination of Australian press representations of SRC in the latter half of the twentieth century, and future research should take up this challenge. However, this unavoidable restriction does present some benefits. Trove's coverage aligns with the period in which Australian newspapers were at their most widely read and arguably most influential. Mayer notes the “immense influence” of the Australian newspaper press throughout the late nineteenth century, and cites a contemporary source who believed the newspaper press “monopolizes the greater part of the thought (of Australians) (Mayer, 1964, p. 16).” According to Goot, this influence diminished in the latter half of the twentieth century. Despite a surge in readership during and directly following the Second World War, Australian newspaper circulation began a steady decline after 1954 (Goot, 1979, p. 7–9). The correlation between the 1954 copyright watershed and the decline of Australian newspaper circulation is a coincidence, but it is a happy one from an epistemological perspective because it means that the sources in the Trove newspaper archive are representative of a golden era in Australian print journalism.

There are also topic-specific advantages to examining newspaper representations of SRC before 1954. Prior to the 1950s, medical knowledge of concussion, and SRC more specifically, was still under development. Casper argues that “by the 1950s, the state of the science was clear regarding risks of repeated concussions (Casper, 2018, p. 805).” Prior to this, from the late nineteenth century onward, pioneering clinicians and researchers were attempting to inform the public that “… CHI [closed head injuries] were dangerous, that repeated concussions should be avoided, and that such injuries brought risks of long-term consequences …. (Casper, 2018, p. 796)” By analyzing newspaper articles published between 1803 and 1954, this study is able to examine how these growing concerns about SRC were transmitted to the Australian public by its most influential source of news media.

To engage fully with the breadth and scope of publications in the Trove archive, I employed a distant reading approach. Distant reading is a quantitative methodology popularized by literary scholar Franco Moretti, which has recently been adopted by other humanities disciplines, including historians (Moretti, 2013, p. 48). Its purpose is to visualize quantitative trends or patterns within large collections of text *via* graphs, maps, or trees. Such an approach is particularly valuable when applied to newspaper collections, which often contain many thousands or millions of distinct texts. Historians and other humanities scholars have been cautious about employing quantitative methodologies like distant reading, and there are ongoing debates about the opportunities and challenges it poses—especially regarding the reductionist tendencies of graph-based analyses (Townsend et al., 2019). In consideration of these concerns, this study follows the lead of recent historical projects that have successfully married distant with a close reading by using graphical trends and patterns to guide the traditional close reading of primary sources^16^.

In this case, I used Trove's inbuilt search capabilities to locate the Australian newspaper articles that contained the word: “concussion.” For the sake of expediency, this study predominantly confines itself to a single search term: concussion. This is an effective search term because (as will become apparent in later sections) its usage in the Australian newspapers encompassed both the immediate and ongoing effects of a sporting brain injury. “Concussion” was also not linguistically specific to a particular activity or sport. As such, I could be confident that the distant reading graph in Figure 2 and subsequent close readings were capturing a range of historical brain injury experiences. However, “concussion” does not necessarily capture all the nuanced ways that sporting brain injuries were represented in the Australian sporting press. There are dozens of other terms used by sports journalists and athletes to describe brain injuries. Future studies may consider employing the terms listed in Table 1^17^.

**Table 1:** Historical terms used by Australian and international journalists to describe sporting brain injuries.

**Immediate effects**	**Prolonged effects**
“Bell ring” or “bell ringer” or “bell rung”	“Boxer's waltz”
“Brain injury”	“Brain fog”
“Brain jarred”	“Can't get in”
“Concussion” or “concussion of the brain”	“Cuckoo”
“Dazed”	“Cutting out paper dolls”
“Head collision”	“Dementia pugilistica” or “boxer's encephalopathy” or “encephalopathy pugilistica”
“Head knock” or “head injury”	“Fighting on their nerves”
“Knock out” or “knockout” or “K.O.”	“Goofy”
“Loss of consciousness”	“Lunacy”
“Paralysis of consciousness”	“Mental collapse”
“Senseless”	“Punchy” or “punch drunk” or “punched silly”
	“Rattle brained”
	“Slap happy”
	“Slug nutty” or “slugnutty”
	“Stumblebum” or “stumbleback”
	“Walking on/off their heels”

I began with a search for “concussion” in all 1,629 newspapers contained in the Trove archive. This search found 346,859 articles published between 1803 and 1954. I recorded the number of ‘concussion' articles published each month and then created a line graph that mapped the publication frequency over time. This initial distant reading was unsuccessful. The visualization was too broad (or too distant) to be useful because it counted ‘concussion' articles in general interest publications and there was no guarantee that the trends and patterns on the graph were directly related to *sport* related concussion. To focus the process, I narrowed the search to the 22 sporting newspapers contained in the Trove digital archive (see Table 2); a strategy employed by historian Gary Osmond to study historical traces of homophobic language in Australian sports journalism (Osmond, 2015). This collection includes publications from cities in every state and territory (except the Australian Capital Territory) but is concentrated in Sydney and Melbourne. This is to be expected, given that Sydney and Melbourne were the cultural, economic, and sporting capitals of Australia throughout much of the previous two centuries. The publications within this collection varied in their coverage but were predominantly focused on sport and leisure. Some carried reporting and opinion on multiple sporting and leisure pursuits while others were concerned with specific activities like horse racing or hunting.

**Table 2:** List of Australian sporting newspapers in the Trove archive, in descending alphabetical order.

**Title**	**Dates published**	**Location**
Arrow	1916–1933	Sydney, New South Wales
The Arrow	1896–1912	Sydney, New South Wales
The Australian Sportsman	1848	Sydney, New South Wales
Bell's Life in Sydney and Sporting Reviewer	1845–1860	Sydney, New South Wales
Bell's Life in Sydney and Sporting Chronicle	1860–1870	Sydney, New South Wales
Bell's Life in Tasmania	1859	Hobart, Tasmania
Bell's Life in Victoria and Sporting Chronicle	1857–1868	Melbourne, Victoria
Bird O' Freedom	1891–1896	Sydney, New South Wales
Call and WA Sportsman	1918–1920	Perth, Western Australia
The Northern Sportsman	1928	Innisfail, Queensland
Referee	1886–1939	Sydney, New South Wales
The Satirist and Sporting Chronicle	1843	Sydney, New South Wales
Saturday Referee and the Arrow	1912–1916	Sydney, New South Wales
Sport	1911–1948	Adelaide, South Australia
Sportsman	1882–1904	Melbourne, Victoria
Sporting Globe	1922–1954	Melbourne, Victoria
Sporting Judge	1914–1918	Melbourne, Victoria
Sporting Life: Dryblower's Journal	1905–1906	Kalgoorlie, Western Australia
Sydney Sportsman	1900–1954	Surry Hills, New South Wales
W.A. Sportsman	1914–1918	Perth, Western Australia
The W.A. Sportsman	1901–1902	Kalgoorlie, Western Australia
Winner	1914–1917	Melbourne, Victoria

**Figure 1:**
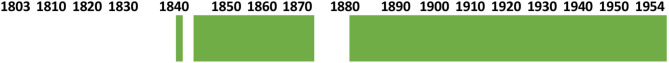
Trove's combined collection of Australian sporting newspapers covers most of the late nineteenth century and the entire first half of the twentieth century. Years in which a sporting newspaper was being published are shaded green.

The longevity of these newspapers was variable. Some, like *The Referee* (1886–1932) ran for several decades while others like *The Northern Sportsman* (1928) were in print for less than a year. Taken together, Trove's combined assortment of sporting newspapers stretches from 1843 to 1954, except for gaps in 1803–1842, 1844, and 1871–1881 (see Figure 1).

**Figure 2:**
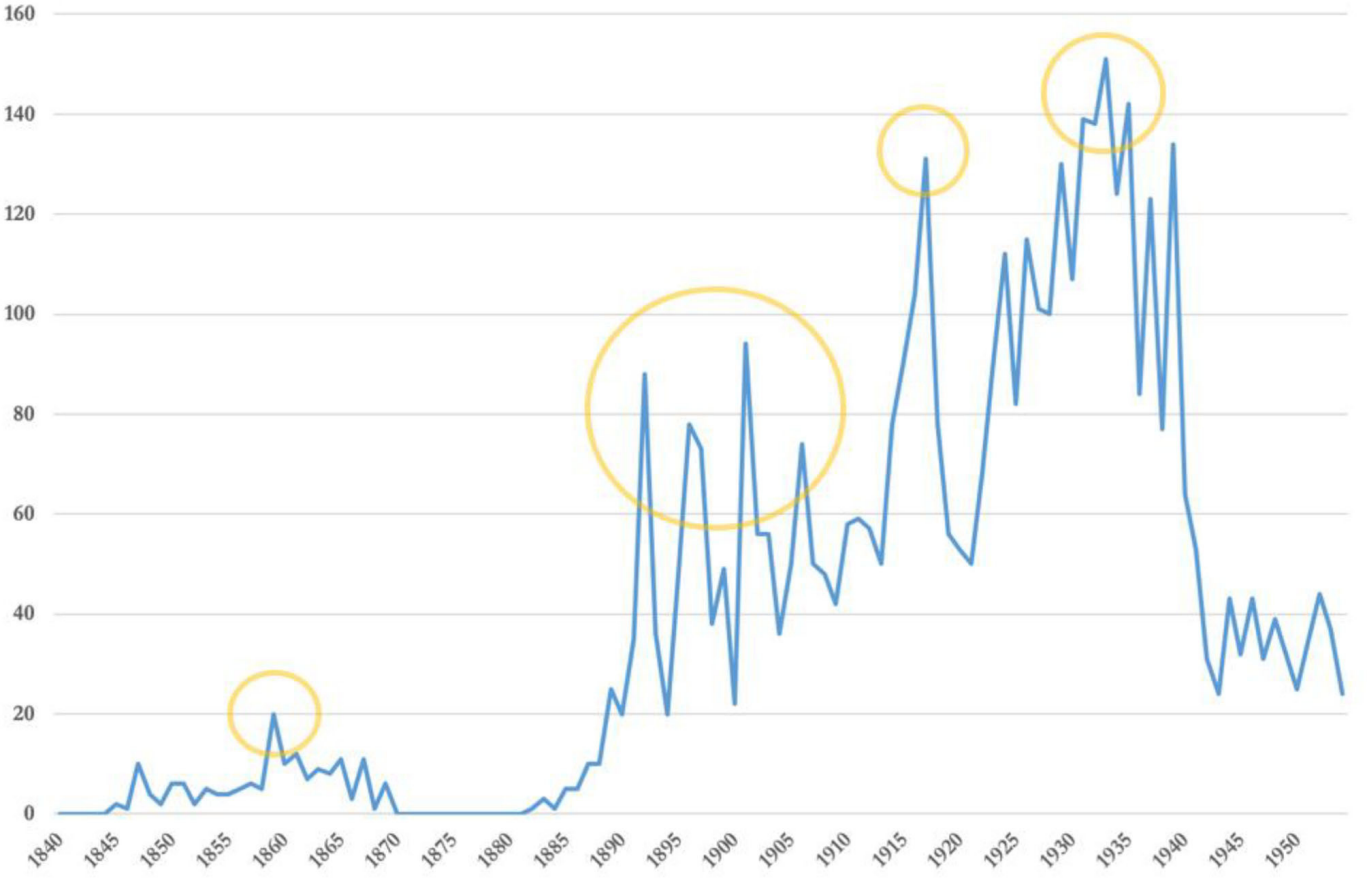
Articles containing “concussion” in 22 Australian sporting newspapers, 1843–1954.

There were 4,671 “concussion” articles published in Australian sporting newspapers between 1843 and 1954^18^. The earliest sporting newspaper in this collection, *The Satirist and Sporting Chronicle* (Sydney, NSW, Australia), began its publication in 1843. The frequency with which these articles were published is charted chronologically in Figure 2. Broadly, mentions of “concussion” appear to rise steadily in the Australian sporting press after 1843, reaching a peak in 1933. The rising mentions are punctuated by a number of noticeable peaks in 1859, 1892, 1896, 1901, 1906, 1917, 1931, 1933, and 1935. These peaks can be grouped into four periods of increased coverage that warrant a closer inspection *via* close reading of individual articles: 1859, 1892–1906, 1917, and the early 1930s.

Before diving into the sources, the methodological underpinnings of this study require further consideration. Restricting the scope of this analysis to sporting newspapers gave me increased confidence that the distant reading in Figure 2 was comprised of articles that addressed “concussion” in a sporting and physical activity context. While this decision was necessary from a methodological perspective, it did produce come epistemological limitations. Commentaries about concussion in non-sporting events such warfare, workplaces and motor vehicle accidents are absent from this study, which means potentially important perspectives about the broader knowledge and attitudes toward brain injuries in Australia are missing. The perspectives of women and Aboriginal and Torres Strait Islander Peoples are also noticeable silences, as sporting newspapers during the nineteenth and early twentieth century focused almost exclusively on the exploits of white, Anglo-Australian men. This step also excluded reports from mainstream journalists who also wrote about sport in substantive and meaningful ways. Acknowledging these limitations speaks of the realities of doing historical research in the “infinite” digital archive. The employment of quantitative methodologies like distant reading allows scholars to engage with a greater range of the newly available digital sources than would otherwise be possible. However, even with the adoption of these methods, individual historians are still required to draw boundaries around their analysis for the sake of expediency or feasibility.

Distant reading presents historians with an epistemic trade-off: context for scale. This approach enabled me to engage a far greater number of sources than would otherwise be possible and provided a helpful visualization to guide my analysis. Despite this, it was still not possible to inspect all areas of potential interest highlighted by the distant reading graph. This study focuses on the peaks of Figure 2 because these features presumably represent historical instances of increased sporting newspaper interest in SRC. However, there are valid reasons for investigating the other features of this graph. Studying the troughs could reveal insights about periods of depressed interest in SRC, and examining the up or down slopes of the peaks may help us better understand the building or declining interest in concussion. Future research should examine these trends, but expediency dictated that this study focuses on a limited but targeted selection of sources. Distant reading may provide a historical map, but we cannot visit everywhere. Herein lies the contradiction of the method. It offers historians a tantalizingly broad view of the available texts but, in the end, only a small selection of sources can be analyzed in greater depth. This produces a vignette-style history, which is revelatory and meaningful in many ways, but is not a comprehensive narrative of Australian press attitudes toward sports concussion.

There are also rhetorical dimensions to consider because the interpretation of a graph can be influenced by its visual arrangement. For example, the visual effect of the 1859 peak may be exaggerated by Trove's 1871–1881 gap in coverage. Similarly, the 1930s peak may also seem more visually striking because it is contrasted with a sharp decrease in “concussion” mentions during the next decade, which was likely to be exacerbated by a dramatic decline in the number of sporting newspapers being published during and following the Second World War (Willmot, 1994). These epistemological and rhetorical factors are significant but they do not diminish the utility of distant reading in historical research. Rather, these considerations reinforce the importance of marrying distant reading with close reading (Townsend et al., 2018a,b). Distant reading is a useful tool for posing new questions about a topic but it is not able to answer them. For example, this graph prompts queries about whether or not Australia experienced a series of early “concussion crises” throughout the nineteenth and twentieth century, which would significantly predate the current crisis. The increased concussion discourse in the Australian sporting press in 1859 long precedes even the 1890s college football concussion crisis identified by Harrison. Establishing the historicity of these potential early crises and understanding the reasons for their development requires close reading and analysis of the contemporary sources.

## Close Reading “Concussion” in Australian Sporting Newspapers

Whilst distant reading may be new to the humanities, close reading is an established methodological tradition. There are many ways to conduct a close reading, and literary scholar David James argues that the method might better be understood as “a complex of ambitions and passions rather than as a definitive, consistent, and systematized procedure (James, 2020, p. 15).” In all cases, close reading involves the methodical and granular exegesis of individual texts, with the aim of revealing how language conveys topic-specific ideas and information to readers. The articles from each of the four periods identified in Figure 2 were analyzed *via* close reading. All articles from these periods were subject to an initial superficial reading, with the articles that were substantively concerned with SRC being selected for a closer examination^19^.

### Dazed and Confused: 1859

Despite a notable 1859 peak on the distant reading graph (see Figure 2), a close reading of the articles published in this year does not confirm the existence of a mid-Victorian concussion crisis in Australia. The press was reporting occurrences of head injuries in sport but there is no indication that journalists in 1859 believed these injuries represented a systemic problem. Analyzing articles published in this period is complicated by variations in the ways that Australian sports journalists used the term “concussion” in 1859. Of the 20 “concussion” articles published in 1859, only 10 specifically referred to head injuries in sport. These were predominantly incidents in boxing or horse racing, and the correspondents described “concussion” as a momentary impact rather than an ongoing condition. For example, an account of a bare-knuckle fight in Melbourne described the moment one of the combatants “went down partly from a slip and partly from the force of the concussion^20^.” An earlier fight story carried a similar description, in which a knock down was not deemed legitimate because it was “partly from the concussion^21^.” In these accounts, concussion refers to the force of an impact itself rather than its cognitive aftereffects.

This understanding of concussion matches other contemporary uses of the term, which were often unrelated to head injuries. Readers of Australian sporting newspapers in 1859 would have seen “concussion” used to describe an exploding artillery shell, a horse's hoof striking the ground, the effect of steam being expelled from a locomotive, and the impact of a wayward hot-air balloon crashing into chimneys along Philip Street in Sydney^22^. These descriptions are linguistically illustrative of the physical impact of a concussion—the brain is subjected to shock or commotion. However, these uses of the word do not align with later conceptions of concussion as a cognitive condition. There were some early signs of more brain-specific uses of the term in 1859. In four other articles published in 1859, “concussion of the brain” is given as a cause of death or debilitation after riders fell from their horses while racing or hunting^23^. The presence of the qualifier ‘of the brain' indicates that the meaning of ‘concussion' was not yet fixed and it would be some time before it was discarded. However, these few references also indicate that a limited number of Australian sports journalists in 1859 recognized that brain injuries could have serious health consequences for athletes.

### A New Century: 1901 and 1906

A closer reading of articles from this period reveals that 1892 and 1896 may have been false peaks (see Figure 2). Approximately 40% of the “concussion” references in these 2 years are attributable to a racehorse by that name and are not related to brain injuries. The popularity of this racehorse, likely named for the thunder of its hooves when it ran, indicates a measure of continued ambiguity in the meaning of “concussion.” Excluding these articles from the data in Figure 2 flattens the spikes in 1892 and 1886 to such an extent that they cannot be considered genuine peaks in press mentions of “concussion.” The aforementioned racehorse does not have the same presence in 1901 or 1906, and the articles published during these years warrant closer scrutiny. Close reading reveals significant changes to Australian journalistic attitudes in 1901 and 1906 when compared with the articles from 1859. Perhaps, the most obvious change is more semantic consistency in the use of “concussion.” In 1901 and 1906, concussion was almost exclusively referred to as “concussion of the brain.” For example, in 1901 the *Referee* declared the misfortune of New South Wales footballer T. Costello, who received “another kick on the head (which) gave him concussion of the brain,” and in 1906 the *Sydney Sportsman* wrote of a young jockey who “sustained concussion of the brain and died the same evening^24^.” As previously, the presence of this qualifier does indicate some continued variability in the meanings ascribed to “concussion.” However, it is noteworthy that, in 1901 and 1906, only 5% of the articles identified referred to concussion in a non-brain-injury context. This suggests that at the turn of the twentieth century, Australian sports journalists strongly associated “concussion” with brain injuries and used it less frequently in other contexts.

The Australian sporting press' increased interest in brain injuries is evident in its reporting on specific incidents during 1901 and 1906. In contrast to 1859, when references to concussion in the Australian sporting press were sparse and procedural, in the first decade of the twentieth century Australian sports journalists were writing more extensively and more critically about brain injuries. This is particularly obvious in reporting on the death of boxer Otto Cribb following a bout in Sydney on July 22, 1901. Cribb, a New Zealander who made his name fighting in Australia, was a renowned sporting figure and his death attracted significant media attention. After being beaten by Mick Dunn in front of a large crowd at the Gaiety Athletic Club, Cribb left the venue just after 10 p.m. and appeared in good health^25^. He then collapsed on the tram ride back to his Bondi hotel room after which his condition deteriorated rapidly. On his return to the room, he complained of a severe headache and was attended to by his trainers and seconds, who said the boxer was delirious as they undressed him and sponged his face with whiskey and water^26^. Around 3 a.m., Cribb's trainer found him cold and unresponsive and announced to his friends and supporters, “I think poor Otto's gone (see text footnote 26).” The attending doctor determined that Cribb had died around 1 a.m. from “concussion of the brain” and a subsequent postmortem confirmed this diagnosis, adding the presence of a “concussion hemorrhage (see text footnote 26).”

The post-fight analysis in the press tried to make sense of how a 23-year-old “noted for his sturdiness” could have died after a fight that displayed an “almost total absence of hard blows^27^.” Press commentary intensified when it was revealed that all involved in the fight had been ordered to appear before a coronial inquest into the death^28^. Sydney's most influential sporting newspaper, the *Referee*, showed a particular interest in the case. Even before the inquest, the newspaper preempted the verdict and showed the extent of its knowledge about concussion:

it is an admitted scientific fact that the outcomes of a concussion of the brain may not become apparent for weeks, months, or even years after the injury has been sustained^29^.

This statement was intended to argue, on behalf of the indicted trainers and officials, that Cribb's death might have been caused by injuries sustained during his recent tour of the USA, rather than on 22 July. Regardless of its purpose, this assertion contrasts markedly with mid-nineteenth century perceptions of “concussion” as an impact causing near instant death or disablement. The next issue of the *Referee* carried a nearly 4,000-word report on the inquest, which contained a visceral account of the postmortem examination of Cribb's brain and also the medical examiner's conclusions on what caused his death:

The concussion would have been caused by a blow on the face. A concussion hemorrhage was produced as follows:—If a blow was received on the front part of the head, the force was transmitted through the semi-solid substance of the brain as through water, and the wave striking against the opposing surface there produced the damage^30^.

By 1901, readers of the Australian sporting press were being provided with information about concussion that was scientifically detailed and included many features recognizable in modern discourse about concussion: it can be fatal; its effects can be delayed; it can be caused by seemingly innocuous incidents; and it can affect “well-nourished and muscular” young athletes (see text footnote 30).

There was also a clear message that concussion was not confined to boxing. Interest in Cribb's death re-intensified in August when the Crown Prosecutor, unsatisfied with the coroner's findings, charged the officials and trainers with felonious killing^31^. Advocating for the accused, the *Sydney Sportsman* argued that boxing was no more or less dangerous than other popular sports, stating that “a fall at football on the back of the head might produce a similar result.” The paper also highlighted remarks from the defendants' lawyer who exclaimed that he “could not say how many deaths have resulted in the last 5 years from boxing contests or from football matches^32^.” “Interstate reporting on the case also noted that death and debilitation from SRC occurred not just in boxing. Melbourne's Sportsman stated ‘there was a risk and danger in all manly sports, such as horseracing, football, cricket, boxing etc., and accidents frequently happened^33^.'” These comments indicate not only that Australian sports journalists acknowledged the possibility of concussion occurring in sports other than boxing but also hint, through their gendered language, at the historical intersections of masculinity and sporting injuries.

In this period, a young man's willingness to risk his health during organized games was a marker of his virility and masculinity, and an indication that he might be willing to further endanger himself in the service of his country, empire, and the white race (Crotty, 2003). The relationship between brain injuries, sport, and gender in Australian history warrants a deeper analysis than can be provided here. However, it is important to note that the Australian sporting press' valorization of concussion was implicit rather than explicit during this period. There are, for example, no statements in either 1901 or 1906 lauding the heroism of athletes who sustained a concussion. However, concussion was clearly accepted as a normal part of “manly” Australian sport, and the sporting press generally viewed brain injuries as a routine part of masculine endeavor. This acceptance of concussion as a normal part of (white) men's sport is a powerful form of implicit celebration.

The general acceptance of concussion as a normal function of men's sport was perforated by discussions about the potentially serious consequences of sporting brain injury, which stimulated institutional and structural critiques, particularly of the horse racing industry. A 1906 article in Western Australia's *Dryblower's Journal* bemoans Australia's “truly appalling” safety record in horse racing, noting a “long list of fatal accidents” that included “no fewer than 35 lads (who) were done to death through sustaining falls in races^34^.” The paper suggests a number of measures to reduce these deaths, including “the ADOPTION OF SKULL PROTECTORS” which it believed would ensure that “concussion of the brain … would be seldom heard of, as they are continually nowadays^35^.” Calls for better protective equipment for jockeys were also being made on the east coast. The *Referee* approved of the Victorian Racing Club's decision in 1906 to enforce the wearing of “stiff skull caps” by jockeys, which the paper believed to be the first example of such a rule anywhere in the world^36^. Critiques of horse racing extended beyond the need for better protective equipment. The *Sydney Sportsman* drew attention to the poor condition of a track at Wyalong following the death of a young jockey from “concussion of the brain^37^.” The paper railed against the owners of the “boy murdering” course and suggested that they cared more about “pushing the sale of gaudily printed programs” than maintaining the turf (see text footnote 37). The *Sportsman* also took issue with the facilities at Kensington racetrack after another concussion death late in 1906, noting that a jockey was “allowed to remain in the ambulance all the afternoon” following a fall^38^. The *Sportsman*'s analysis echoed *Dryblower's Journal* earlier in the year when it called for “the provision of thoroughly equipped casualty rooms on all racecourses^39^.”

The coverage of Otto Cribb's death in 1901 and critiques of the horse racing industry in 1906 illustrate a marked shift in attitudes toward concussion since the mid-nineteenth century. Australian sports journalists moved from referring to concussion as isolated accidents in 1859, to providing extensive commentary on the neurological and institutional considerations of ‘concussion of the brain' in sport and leisure during the first decade of the 20th century. It is clear that columnists in 1901 and 1906 saw the risk of death and disablement from brain injuries as part of the masculine fabric of sport and leisure activities. It is equally clear, however, that the Australian sporting press was communicating to its readers a sophisticated level of knowledge about concussion, and was beginning to acknowledge that concussion was a systemic issue in sport and leisure. Although there were repeated concerns about concussion in the sporting press, the commentary does not indicate that journalists believed these concerns might bring about fundamental changes to Australian sports. The criticism of horse racing, for example, focused on the addition of protective equipment and better policing of corrupt or incompetent course officials. These were undoubtedly valid critiques, but they suggest that the causes of concussion were supplementary rather than fundamental to the practice of horse racing. In the writing of the Australian sporting press, horse racing itself was not fundamentally dangerous because it could be made safe for Australian riders with relatively minor alterations. The twenty-first century concussion crisis, by comparison, has prompted many Australians to see concussion as an inherent risk of some activities and to consider whether this makes these sports too risky to play. Malcolm argues that this type of axiological or existential re-evaluation is one of the key features of a concussion “crisis^40^.” The absence of axiological debate in the articles about concussion published in 1901 and 1906 is an important distinction and, as such, it is premature to claim that Australia experienced a concussion crisis in the early 1900s.

### The Home Front: 1917

The dramatic spike in mentions of “concussion” during 1917 (see Figure 2) was dominated by horse racing articles. More than 80% of the 131 “concussion” articles published in sporting newspapers were concerned with horse racing. Of these, 30 are the results for yet another racehorse with an inconvenient name. The dominance of horseracing in this collection of articles is not surprising given the broader context of the time^41^. Horse racing was arguably the most popular spectator sport in Australia during the early twentieth century, especially among the working class (Fowler, 2019). In 1917, it was also one of the few sporting activities still operating. Australia was reeling from immense losses of people and resources after nearly 3 years of involvement in the First World War. Most sporting activities had either ceased or been significantly curtailed, with the notable exceptions of horse racing and boxing^42^. Horse racing continued relatively unabated until September 1917, when the national War Precautions (Control of Sport) Regulations 1917 were introduced under the *War Precautions Act 1914–1916* in an attempt to “refocus the nation's energies away from distractions” and to aid military recruitment^43^. Horse racing was therefore one of the only activities on which sports journalists could report throughout much of 1917 and this, combined with the fact that concussions were a relatively common injury for jockeys, goes some way to explaining its dominance that year.

Mentions of concussion in 1917 did not exhibit the same hallmarks of concern that were present in articles published in 1901 or 1906. There are several mentions of jockeys sustaining concussion after falling from their mounts, and a notable number of these injuries were serious. Les Griffiths “sustained severe concussion of the brain” racing in Dubbo and was “unconscious from Wednesday until Saturday,” John Smithson “was thrown and died a few days later from concussion,” and S. Griffiths “received concussion of the brain and did not regain consciousness^44^.” Although these accounts describe the potentially serious consequences of concussion, there were few attempts to interrogate the causes of these incidents or to place them within an institutional context. The *Sydney Sportsman* listed “concussion of the brain” among the list of injuries suffered by riders when calling for a reform of the Australian Jockey Club's compensation scheme, and also noted the “inadequacy of ambulance arrangements” following a major accident at Moorefield racecourse^45^. The death of boxer Sid Lorraine in April was described at some length in the *Referee*, but the paper argued that a 20-year old's death was “one of those unfortunate accidents, which will happen, no matter how the ingenuity of man might be employed to prevent them^46^.” The concerns that were beginning to coalesce in 1901 and 1906 appear to have dissipated, and in 1917 concussion was being reported in a procedural and sometimes dismissive fashion. The factors underpinning this shift require further investigation but the influence of the First World War cannot be overstated. Newspapers were still reporting on horse racing (and boxing to a more limited extent), and incidents of sports concussion were still significant enough to draw media attention. However, with the nation's attention focused on the carnage of the European and Middle Eastern battlefields, the sporting press showed little motivation to critique the systemic factors that underpinned ‘concussion of the brain' in the few domestic sports still being played.

### Punch Drunk in the Early 1930s: 1931, 1933, and 1935

Mentions of concussion in Australian sporting newspapers reached their highest level during the early 1930s, with peaks in 1931, 1933, and 1935 (see Figure 2). There is an immediately noticeable linguistic change compared with earlier reporting. By 1931 “concussion of the brain” was rarely used and was replaced in almost every instance by “concussion,” indicating that this word was now so strongly identifiable with brain injuries that there was no need to insert a qualifier. Among the 432 articles that contained “concussion” during 1931, 1933, and 1935, there were reports of concussion in a wide range of sporting activities, including Australian football, cycling, horse racing, motor racing, cricket, rugby football, association football (soccer), and even skiing. However, the most substantive and urgent commentary on concussion took place in articles about boxing. Australian sporting newspapers had been reporting critically on boxing concussions since the turn of the twentieth century. In 1901 and 1906, journalists conveyed to their readers a sophisticated understanding of concussion's neurological underpinnings, and also exhibited an evolving understanding of the institutional factors that led athletes to sustain brain injuries. Critical commentary on concussion was subdued during the First World War but re-intensified in the early 1930s, coalescing around concerns for Australian boxers.

Initial signs of these reignited fears can be found deeper within a meandering piece published in the *Referee* in 1931, in which long-retired fighter Edward “Hock” Johnson offered his views on the current state of boxing. Johnson told correspondent Jack Elliot that he wanted a return to bare-knuckle fighting because it would reduce the number of fighters “tottering about the street ‘punch drunk^47^.”' Elliot repeated these concerns a few years later, telling his readers that “bare-knuckle battlers never suffered from it^48^.” Melbourne's *Sporting Globe* also sounded the alarm, twice reprinting an instructional article that warned young boxers: “a number of severe knockouts disturb the brain so seriously as to bring on lunacy^49^.” In 1933, the *Sydney Sportsman* called for a reform to medical examinations of boxers. The paper argued that the current examinations were too cursory and that “any boxer with a weak cerebral system should be barred from entering the ring. The risk is too great^50^.” Perhaps the most vivid expression of these intensifying concerns about concussion came from the famous American heavyweight Gene Tunney. In 1933, Tunney told the *Sporting Globe*'s readers about a concussion he sustained during a 1927 sparring session, after which he spent 3 days in “a state of returning consciousness” and quickly realized “I had a concussion (see text footnote 50).” He suffered memory loss and paranoia, and described a sensation that was “as though hot water had been poured through a hole in my skull and flowed down over my brain to my eyes leaving a hot film (see text footnote 50).” The experience hastened his retirement the following year because “any sport in which such accidents could happen was dangerous … the possibility of becoming punch drunk haunted me for weeks (see text footnote 50).” Although it is hard to be certain about any audience reaction to an historical text, it is reasonable to assume that readers of Tunney's visceral account would have been prompted to consider not only the immediate agony of concussion but also its potential to bring about long-term consequences. The specter of these lasting consequences had been present in reporting throughout the late nineteenth and early twentieth century but by the 1930s the Australian sporting press could put a name to it: “punch drunk.”

Several mentions of “punch drunk” emerged during my close reading of “concussion” articles and, to study the usage of this term more fully, I conducted a search for it in isolation. Methodologically, this step is an example of the nonlinear nature of distant reading. In theory, distant reading leads to close reading. In practice, however, the process is far more iterative, with the researcher constantly toggling between distant and close perspectives. In this case, the process moved from distant reading to close reading to distant reading and back to close reading again, with several detours along the way. There were 247 articles containing “punch drunk” in the Australian sporting press between 1843 and 1954, and the majority of these were published in 1930s. The first relevant mention of the term was in 1918 where it was used to describe a transient state of disorientation immediately following a hard blow to the head^51^. In this context it was semantically indistinguishable from “concussion” but this changed after 1928, when Australian sports journalists began referring to “punch drunk” as a debilitative and permanent neurological condition. This shift was undoubtedly influenced by Harrison Martland's highly publicized 1928 study of “degenerative, progressive dementia with Parkinsonian-like features” that was common among the deceased boxers in the USA (Casper, 2018, p. 804). This international impetus is unsurprising because, prior to the Second World War, Australian neuroscience was predominantly guided by outside actors. Local clinicians and researchers produced “opportunistic” studies of brain disease and injuries but there was a “heavy dependence on British, and to a lesser extent Western European and American medicine (Eadie, 2000, p. 4).” This persisted until the clinical neurology was formally established as a profession in Australia during the 1930 and 1940s. Martland's findings, therefore, had a receptive audience among Australian medical professionals, clinicians, and his use of the colloquial term “punch drunk” also ensured that his research could be easily relayed to the Australian public. Adelaide's *Sport* newspaper provided extensive commentary:

…nearly one-half of the fighters who have stayed in the game long enough develop this condition, either in a mild form or a severe and progressive form … the condition can no longer be ignored^52^.

During the early 1930s, “punch drunk” appeared frequently in the articles that drew attention to the potential consequences of prolonged involvement in boxing.

Australian sporting press discourse about SRC in boxing during the 1930s differed from that of previous eras because it was consistent, impassioned, and called for fundamental changes in the conduct of the sport. Sydney's Referee led the debate by publishing editorials that called for greater awareness of the long-term consequences of repeated concussions and implored boxers to better protect themselves, either by changing their technique or retiring earlier. In January 1933, the paper asked its readers “Are Our Fighters Becoming Punch Drunk?” before noting that “many of them are punch drunk before they are out of their teens^53^.” The *Referee* laid the blame at the feet of promoters and trainers, who were exposing poorly trained fighters to danger “for the sake of a small, but an immediate, box office profit (see text footnote 53).” The *Sydney Sportsman* lamented “punch drunk and forgotten” fighters who had been discarded by the industry after years of punishment and called upon the current crop of young pugilists to be aware that their brain health was being sacrificed for profit: “they are doing it now—and do not realize it^54^.”

There was also an emerging opinion in the sporting press that a boxer's overall exposure to repeated blows, rather than their ability to “take a punch,” was the deciding factor in whether or not they developed punch drunk syndrome. The *Referee*'s Jack Elliot, noted that the disease made no accommodations for age, experience, or physical hardiness:

You find them punch drunk after years of fighting, and you find them punch drunk as kids 18 or so … it even attacks the steel jawed … punch drunks do not make old bones^55^.

Elliot was a former Olympic boxer who had retired in 1931 due to concussion, and he wrote passionately about the “tragedy of the ‘punch drunk' (see text footnote 55).” He prompted his readers to question their perceptions of boxing as a healthy pursuit when he noted that athletes in otherwise superb physical condition were prone to sudden and often inexplicable deaths: “these hardy Spartans fade easily from life^56^.” He also noted that health concerns were turning public opinion against boxing: “the publicity given the half a dozen fighting deaths over the past 2 years have done severe damage to the sport (see text footnote 56).”

This critical commentary in the sporting press corresponds closely with a dramatic shortening in Australian boxing careers, as noted by neuropsychologist Clausen et al.:

Since the 1930s, the average duration of a professional boxer's career has dropped from 19 years to 5 years, and the mean number of career bouts has reduced from 336 to 13. This is despite no significant decline in participation rates from 1931 until 2002 (Clausen et al., 2005).

The close correlation between intensified press reporting on punch drunkenness and the beginning of a significant decline in the career longevity of boxers is sufficient to suggest a relationship between the two events. This relationship requires further investigation because given the span of time over which this change occurred, the shortening of Australian boxing careers was undoubtedly multifactorial and cannot be attributed solely to the influence of the press. However, evidence from sporting newspapers suggests that press reporting during the early 1930s about the dangers of concussion may have prompted Australian boxers to shorten their careers, in the hope of avoiding punch drunkenness. This type of axiological evaluation in which health risks are balanced against the benefits of sporting participation is a key feature of the current concussion crisis, and the preliminary evidence presented here is sufficient to suggest that Australia experienced a limited concussion crisis during the early 1930s, confined to boxing.

## Conclusion

Distant reading identified four distinct periods of elevated usage of ‘concussion' in the Australian sporting press: 1859, the early 1900s, 1917, and the early 1930s. The articles published during these periods were scientifically well-informed, critical of the systemic and institutional issues underpinning the exposure to SRC, and called for changes to the conduct of Australian sports. Two of these periods contained particularly critical commentary on SRC in Australian sport. Articles published during the early 1900s and the early 1930s showed discursive similarities with the current debates about SRC in sport. In the early 1900s, the death of boxer Otto Cribb generated significant press interest in the delayed effects of concussion and reporting on the subsequent trial showed that the Australian sporting press in 1901 possessed a sophisticated understanding of SRC pathology. Critiques of the horse racing industry in 1906 also indicated that the sporting press was aware that SRC was linked to institutional issues in sport. In the early 1930s, concerns about SRC in boxing manifested as a punch-drunk-crisis, which contributed to structural changes in Australian boxing. This evidence should be regarded as illustrative rather than conclusive, and further research on this topic remains to be done. This distant reading and the close readings born from it are slices in time taken from a specific arm of the Australian media. Future research should further contextualize the findings of this study by linking sporting newspaper representations of concussion to discussions in the broader Australian press, which could be further connected to international concussion discourse. If an effective work-around to the Australian copyright watershed can be found, the evidentiary gap between the end of Trove's coverage in 1954 and the ‘beginning' of the 21st century concussion crisis should also be bridged. This preliminary research does, however, show that Australian sports journalists were transmitting sophisticated neurological knowledge of SRC to their readers as early as 1901, and were also engaged in concerted but contained critiques of the systems and practices underpinning the approaches to brain injuries in sport.

Although there are clear parallels between reporting on concussion in 1901, 1906, and early 1930s, with the twenty-first century concussion crisis, there are also important differences. Concerns about SRC during the early 1900 and 1930s were confined to specific sports, most notably boxing and horse racing. This lack of universality perhaps goes part of the way toward explaining why concerns about SRC did not gather momentum until nearly 160 years after the term first appeared in the Australian sporting newspapers. The twenty-first century concussion crisis was sparked by fears about SRC in American football, but these were quickly extrapolated to a myriad of sports and leisure activities. The same process does not appear to have occurred in the Australian sporting press during the early 1900s and early 1930s. Although SRC was reported in multiple activities throughout the nineteenth and twentieth centuries and journalists did suggest that concussion was a feature of “all manly sports,” there was no discernible attempt in the articles analyzed here to connect the institutional or cultural causes of concussion in one sport to those in another. Unlike the current concussion crisis, systemic critiques of SRC were confined to specific sports, most particularly horse racing and boxing.

The compartmentalized nature of press reporting on concussion during these years reflects the broader state of Australian sports governance throughout much of the twentieth century. The recent concussion crisis in Australian sport is bolstered by advances in scientific knowledge and the ubiquity of globalized media networks. It is also facilitated by a centralized and an interconnected Australian sporting bureaucracy. Although many press reports about concussion during the late nineteenth and early twentieth century were critical, impassioned, and based on the latest available scientific evidence, journalists' concerns struggled to gain traction outside their immediate sporting context. For example, responsibility for addressing fears about punch drunkenness during the 1930s fell to individual fighters, trainers, and promoters. Whilst there is some evidence to suggest that the boxing community acted on these concerns, there is no indication of the articles studied that systemic fears about neurodegenerative diseases spread to other Australian sports.

This is partly because Australian sporting bureaucracies prior to the 1980s were “fragmented and decentralized (Horton, 2015).” It did not take a great leap of scientific knowledge to imagine that concussions sustained in boxing might have the same long-term effects as those sustained in other popular Australian sports, but there was not yet any overarching apparatus for recognizing this potential, funding scientific investigations into it, and coordinating a response across multiple sports and leisure activities. By comparison, the current Australian sports governance model is highly centralized, with taxpayer funded bodies like Sport Australia and the Australian Institute of Sport able to coordinate multisport responses to major issues like concussion^57^. These bodies are further entwined with national medical associations like Sports Medicine Australia (SMA) and the Australian Medical Association (AMA), who link them to a rapidly proliferating body of knowledge about the brain that is being generated not just from studies of concussion but^57^

also Dementia, Alzheimer's, Parkinson's, Amyotrophic Lateral Sclerosis (ALS), and a host of other neurodegenerative diseases. It is this network of sporting and medical bodies that drives nationwide rule and policy changes, and facilitates the production of documents like the 2019 Concussion in Sport Position Statement, which was jointly written by Sport Australia, SMA and the AMA. There are certainly critiques to be leveled at the centralization and bureaucratization of Australian sport and leisure, but these structures provide a framework onto which public concerns about concussion can be grafted. The fragmented nature of Australian sports governance throughout the early twentieth century was such that journalists were often writing into the void when airing concerns about concussion, or at best speaking to a limited, single-sport audience. It is possible that had these interconnected, scientised sporting bureaucracies existed in 1901, 1906, or the early 1930s, the sporting press could have prompted structural changes to how Australian sport deals with concussion.

## Data Availability Statement

The datasets presented in this study can be found in online repositories. The names of the repository/repositories and accession number(s) can be found at: trove.nla.gov.au.

## Author Contributions

The author confirms being the sole contributor of this work and has approved it for publication.

## Conflict of Interest

The author declares that the research was conducted in the absence of any commercial or financial relationships that could be construed as a potential conflict of interest.

## Footnotes

^1^Meanjin is the Australian Aboriginal word for the land on which the city of Brisbane is now located.

^2^Glenn Valencich, “Dual Injury Heartbreak Overshadows AFL Grand Final in Horror Start,' *7 Sport*, 24 October 2020, https://7news.com.au/sport/afl/dual-injury-heartbreak-overshadows-afl-grand-final-after-horror-start-c-1449796.

^3^Peter Ryan and Daniel Cherny, ‘Dangerfield Cleared as Vlastuin Defends Hit,' *The Age*, 25 October 2020, https://www.theage.com.au/sport/afl/disappointed-dangerfield-shrugs-off-vlastuin-incident-20201025-p568d5.html.

^4^This count is based on a Google search for the words “Nick Vlastuin” AND “concussion,” which returned news reports of three previous concussions, including a strikingly similar incident in April 2017. It is possible, even probable, that Vlastuin has suffered further concussions in non-AFL fixtures or training sessions that were not reported in the media.

^5^SRC is referred to by a variety of terms, including: concussion, velocity brain shaking, sports concussion, closed head injury (CHI), mild traumatic brain injury (mTBI). This paper generally uses SRC or concussion but I also employ contemporaneously relevant terms in some instances.

^6^This data are taken from the 2020 Sport Australia AusPlay report, which collected participation data between June 2019 and June 2020. Children are defined in this report as being 14 years and under. For boys, the five most popular contact or combat sports are: Football (soccer), Australian Rules football, Rugby League, Karate, and Rugby Union. For girls: Football (soccer), Australian Rules Football, and Karate. For further information on this data, see: Clearinghouse for Sport, “AusPlay Results,” accessed 7 January 2021: https://www.clearinghouseforsport.gov.au/research/ausplay/results.

^7^Cricket is often assumed to be a slow and genteel game with minimal risk of serious injury. However, in November 2014, Australian batsman Phillip Hughes died on-field after being struck in the base of his skull by a “bouncer:” a type of delivery (pitch) aimed at his head and intended to intimidate. The impact crushed Hughes' vertebral artery, causing a brain hemorrhage. Although Hughes' death was not technically the result of a concussion, it focused public attention on head injuries in cricket. More recent high-profile incidents have further exacerbated public debate about concussions in cricket. During 2019 Ashes series, former national team captain Steve Smith was struck in the head whilst batting and returned to play shortly after, only to fail a concussion test the next day. Hughes' death in 2014 led to widespread debate about helmet design and calls to outlaw the bouncer delivery while Smith's 2019 Ashes concussion resulted in discussions about the appropriateness of current concussion protocols and also raised questions about how a hypermasculine team culture might have encouraged him (and others) to risk their brain health for the sake of the team.

^8^Screen Australia, ‘Television Industry Top Programs, Top 50 Titles on Free-To-Air Television, Metro and Regional, 2008 – 2019' accessed 11 January 2021, https://www.screenaustralia.gov.au/fact-finders/television/industry-trends/top-programs. Data for 2020 were not available at the time of writing.

^9^Data on the incidence of SRC in Australian sport are variable and further research is required. A 2020 systematic review of Australian SRC research found a significant variance in the incidence rates of SRC in the country's three most popular collisions sport codes. For further details, see: Symons et al. (2020).

^10^Australian Government Department of Health, “Medical Research Future Fund, Traumatic Brain Injury Mission: Roadmap,” accessed 8 February 2021, https://consultations.health.gov.au/health-economics-and-research-division/mrff-tbi-mission/.

^11^Emma Kemp, “AFL considering proposal for landmark multimillion-dollar concussion trust for players,” *The Guardian*, 17 February 2021, https://www.theguardian.com/sport/2021/feb/17/afl-considering-proposal-for-landmark-multimillion-dollar-concussion-trust-for-players. See for further information about the Australian Football League's troubled relationship with concussion research: Wendy Carlisle, “The AFL's Concussion Problem,” *The Monthly*, September 2018, https://www.themonthly.com.au/issue/2018/september/1535724000/wendy-carlisle/afl-s-concussion-problem#mtr.

^12^This count of news articles on the Google database should be regarded as indicative rather than conclusive but it does provide a rough measure of the increasing Australian media interest in SRC since 2010: 54 articles; 2011: 85 articles (57% increase); 2012: 77 articles (9% decrease); 2013: 157 articles (103% increase); 2014: 336 articles (114% increase); 2015: 417 articles (24% increase); 2016: 650 articles (55% increase); 2017: 1,060 articles (63% increase); 2018: 1,570 articles (48% increase); 2019: 3,120 articles (103% increase); 2020: 7,120 articles (128% increase). The data were collected on February 9, 2021.

^13^Ken Belson, “The Concussion Crisis in Australian Rules Football,” *New York Times*, 29 September 2019, https://www.nytimes.com/2019/09/26/sports/afl-football-concussions.html.

^14^It was later revealed by the *Sydney Morning Herald* that one of those diagnosed was well-known former NRL player and coach, Steve Folkes. For further details, see: Adrian Proszenko and Adam Pengilly, “Steve Folkes Makes History as First League Player Diagnosed with CTE,” *Sydney Morning Herald*, 14 July 2019, accessed 8 February 2021, https://www.smh.com.au/sport/nrl/steve-folkes-makes-history-as-first-league-player-diagnosed-with-cte-20190713-p526xj.html.

^15^Michelle Blowes, “Brain Disease Found in Former Australian Rules Player,” *The University of Sydney (news and opinion)*, 27 February 2020, accessed 8 February 2021, https://www.sydney.edu.au/news-opinion/news/2020/02/27/brain-disease-found-in-former-australian-rules-player.html.

^16^For examples of distant reading's recent use in historical studies, see: Bode (2018) and Underwood (2019).

^17^This list was compiled by consulting contemporary Australian press reports and international secondary sources—mostly on the history of boxing. As the historical study of sports concussion progresses, this list should be expanded to include more terms, including those used by non-English speakers. For example, Germans often referred to a punch-drunk fighter as “weich birne,” which translates to “soft pear”: (Unterharnscheidt and Taylor-Unterharnscheidt, 2003, p. 543).

^18^It should be noted that this number may include article reprints, either across domestic syndications (for example, the various *Bell's Life* publications) or reprinted from international outlets. Reprinted articles included in the close-reading analysis are indicated as such.

^19^I have avoided using the term SRC in the close reading examinations because this modern acronym jars with the historical terms used in the primary sources. As a result, the less specific but more temporally appropriate “concussion” has been substituted for SRC in the proceeding sections.

^20^*Bell's Life in Victoria and Sporting Chronicle*, 23 July 1859, 3.

^21^*Bell's Life in Victoria and Sporting Chronicle*, 16 April 1859, 2.

^22^*Bell's Life in Sydney and Sporting Reviewer*, 16 April 1859, 4; *Bell's Life in Victoria and Sporting Chronicle*, 10 December 1859, 4; *Bell's Life in Tasmania*, 22 November 1859, 4; *Bell's Life in Sydney and Sporting Reviewer*, 1 January 1859, 3.

^23^*Bell's Life in Sydney and Sporting Reviewer*, 5 March 1859, 4; *Bell's Life in Tasmania*, 12 July 1859, 3; *Bell's Life in Sydney and Sporting Reviewer*, 18 June 1859, 4; *Bell's Life in Sydney and Sporting Reviewer*, 16 July 1859, 4.

^24^*Referee*, 4 September 1901, 8; *Sydney Sportsman*, 31 January 1906, 1.

^25^*Sportsman*, 30 July 1901, 3.

^26^*Referee*, 31 July 1901, 7.

^27^*Referee*, 31 July 1901, 7; *Referee*, 24 July 1901, 7.

^28^*Sydney Sportsman*, 7 August 1901, 2.

^29^*Referee*, 24 July 1901, 7.

^30^*Referee*, 31 July 1901, 7.

^31^The coronial inquest and the criminal case that followed hinged not only on whether or not a concussion killed Otto Cribb but also whether or not the combatants had breached the laws of amateurism. The Crown argued that Cribb and Dunn were competing in an organized prize fight rather than an amateur boxing match. Under the contemporary rules of Australian amateurism, a boxing match was defined as a “display of skill between two friends” while a prize fight occurred when the combatants “intended” to harm one another during the bout. In terms of conforming to the laws of amateurism, the intent of the participants was deemed more important than whether or not they received compensation for their involvement. If it was established that the bout was a prize fight, Dunn and the organizers, trainers, and officials would have been culpable for the injuries that caused Cribb's death. This case is one example of the complexities and contradictions of early 20th century amateurism and warrants further investigation.

^32^*Sydney Sportsman*, 7 August 1901, 2.

^33^*Sportsman*, 8 October 1901, 3.

^34^*Sporting Life: Dryblower's Journal*, 20 January 1906, 5.

^35^*Sporting Life: Dryblower's Journal*, 20 January 1906, 5 (emphasis in original).

^36^*Referee*, 17 January 1906, 6.

^37^*Sydney Sportsman*, 31 January 1906, 1.

^38^*Sydney Sportsman*, 3 October 1906, 1.

^39^*Sporting Life: Dryblower's Journal*, 20 January 1906, 5.

^40^*The Concussion Crisis in Sport*, 2.

^41^The preponderance of reports on horse racing in 1917 was further exacerbated by the fact that some “sporting” newspapers focused predominantly on horse racing. For example, Melbourne's *Sporting Judge* catered primarily to race-going gamblers.

^42^The continuation or cessation of sporting activities (especially boxing) in Australia during the First World War was a source of significant political, social, and even sectarian debate. See for further reading: (Phillips and Moore, 1994).

^43^“Bitter Enemies of the Sporting Fraternity,” 1043.

^44^*Sydney Sportsman*, 21 February 1917, 10; *W.A. Sportsman*, 20 July 1917, 3; *Sydney Sportsman*, 26 September 1917, 5.

^45^*Sydney Sportsman*, 16 May 1917, 4; *Sydney Sportsman*, 15 August 1917, 1; *Referee*, 25 April 1917, 8.

^46^*Referee*, 25 April 1917, 8.

^47^*Referee*, 27 May 1931, 14.

^48^*Referee*, 13 June 1935, 15.

^49^*Sporting Globe*, 15 July 1931, 1; *Sporting Globe*, 25 July 1931, 6.

^50^*Sporting Globe, 17 May 1933, 7*.

^51^*Arrow*, 22 March 1918, 6.

^52^*Sport*, 31 January 1929, 22.

^53^*Referee*, 18 January 1933, 3.

^54^*Sydney Sportsman*, 30 September 1933, 15.

^55^*Referee*, 13 June 1935, 16.

^56^*Referee*, 19 October 1933, 21.

^57^Sport Australia was formerly known as the Australian Sports Commission. The name change occurred in 2018.
